# The Effect of Hydrofluoric Acid Etching Duration on the Surface Micromorphology, Roughness, and Wettability of Dental Ceramics

**DOI:** 10.3390/ijms17060822

**Published:** 2016-05-27

**Authors:** Ravikumar Ramakrishnaiah, Abdulaziz A. Alkheraif, Darshan Devang Divakar, Jukka P. Matinlinna, Pekka K. Vallittu

**Affiliations:** 1 Dental Biomaterials Research Chair, Dental Health Department, College of Applied Medical Sciences, King Saud University, P. O. Box 10219, Riyadh 11433, Kingdom of Saudi Arabia; aalkhuraif@ksu.edu.sa (A.A.A.); darshandevang@gmail.com (D.D.D.); jpmat@hku.hk (J.P.M.); pekval@utu.fi (P.K.V.); 2 Dental Materials Science, Faculty of Dentistry, The University of Hong Kong, Hong Kong, SAR, China; 3 Department of Biomaterials Science, Institute of Dentistry, University of Turku, Turku, FI-20520, Finland; 4 City of Turku, Division of Welfare, Turku FI-20014, Finland

**Keywords:** dental ceramics, lithium disilicate glass ceramics, acid etching, micromorphology, surface roughness, wettability

## Abstract

The current laboratory study is evaluating the effect of hydrofluoric acid etching duration on the surface characteristics of five silica-based glass ceramics. Changes in the pore pattern, crystal structure, roughness, and wettability were compared and evaluated. Seventy-five rectangularly shaped specimens were cut from each material (IPS e-max™, Dentsply Celtra™, Vita Suprinity™, Vita mark II™, and Vita Suprinity FC™); the sectioned samples were finished, polished, and ultrasonically cleaned. Specimens were randomly assigned into study groups: control (no etching) and four experimental groups (20, 40, 80 and 160 s of etching). The etched surfaces’ microstructure including crystal structure, pore pattern, pore depth, and pore width was studied under a scanning electron microscope, and the surface roughness and wettability were analyzed using a non-contact surface profilometer and a contact angle measuring device, respectively. The results were statistically analyzed using one-way analysis of variance (ANOVA) and the *post hoc* Tukey’s test. The results showed a significant change in the pore number, pore pattern, crystal structure, surface roughness, and wettability with increased etching duration. Etching for a short time resulted in small pores, and etching for longer times resulted in wider, irregular grooves. A significant increase in the surface roughness and wettability was observed with an increase in the etching duration. The findings also suggested a strong association between the surface roughness and wettability.

## 1. Introduction

Dental ceramics are biocompatible, natural-looking indirect restorative materials in modern restorative dentistry because of their aesthetic and biomechanical properties [1,2,3]. Ceramics are widely used, and there is a growing interest for their application in various disciplines of dentistry [4,5,6]. Development of computer-aided design/computer-aided machining (CAD/CAM) technology has enabled clinicians to deliver high-quality, precise ceramic restorations [4]. However, the lack of direct chemical bonding with natural enamel and low tensile strength has been a great concern over the years.

In dentistry, adhesion between two dissimilar materials may be enhanced by increasing the surface free energy [6,7]. Increasing the surface free energy improves the wettability of the surface for resin cement bonding [8]. Thus, it is recommended to condition the internal surface of the ceramic restoration with hydrofluoric acid. According to several recent studies, etching of ceramic with 5% hydrofluoric acid for 2–3 min is enough to selectively dissolve the glassy phase [9,10] and make the surface porous for resin composite cement penetration [5,9,11,12]. This porous surface not only provides more surface area for resin bonding [8], but also exposes and generates hydroxyl groups on the ceramic surface that are responsible for chemical bonding via silane coupling agents [9,13,14].

After etching, the ceramic surface is treated with an activated silane coupling agent to improve chemical adhesion [8,15,16,17,18,19,20] and to provide reliable and durable chemical bonding with adhesive resin composite cement [13,21,22,23]. Silane coupling agents (silanes) are hybrid inorganic-organo-functional trialkoxysilane monomers and are capable of unifying organic and inorganic materials. In general, silanes have non-hydrolysable groups (such as methacrylate) and hydrolysable groups (such as ethoxy), which is why they are chemically bifunctional [8]. When reactive silanes are applied over the etched ceramic surface, the hydrolysable alkoxy groups react with exposed hydroxyl groups, and non-hydrolyzable organic groups polymerize with unset resin composite cement [6]. Hence, for reliable and durable chemical bonding, it is necessary that the ceramic surface should be conditioned. However, such chemical reactions are not applicable for non-silica containing zirconia-based ceramics [24].

Numerous studies have reported the effect of acid etching, etching duration, and acid concentration on the surface micromorphology, bond strength, and flexural strength of different ceramics, but none of the studies attempted to correlate altered surface topography to surface wettability. Furthermore, to the author´s knowledge, none of the studies describe in detail the effect of acid etching duration on micro- and nano-scale parameters of the porosities. This is why the current study was aimed to (a) study the effect of acid etching duration on the pore pattern, pore width, and pore depth. and (b) study the effect of etching duration on surface roughness (*S*_a_) and wettability of five selected silica-based glass ceramics.

The null hypothesis tested was that the etching duration has no effect on the micromorphology, surface roughness, or wettability of ceramic surfaces.

## 2. Results

**Figure 1:**
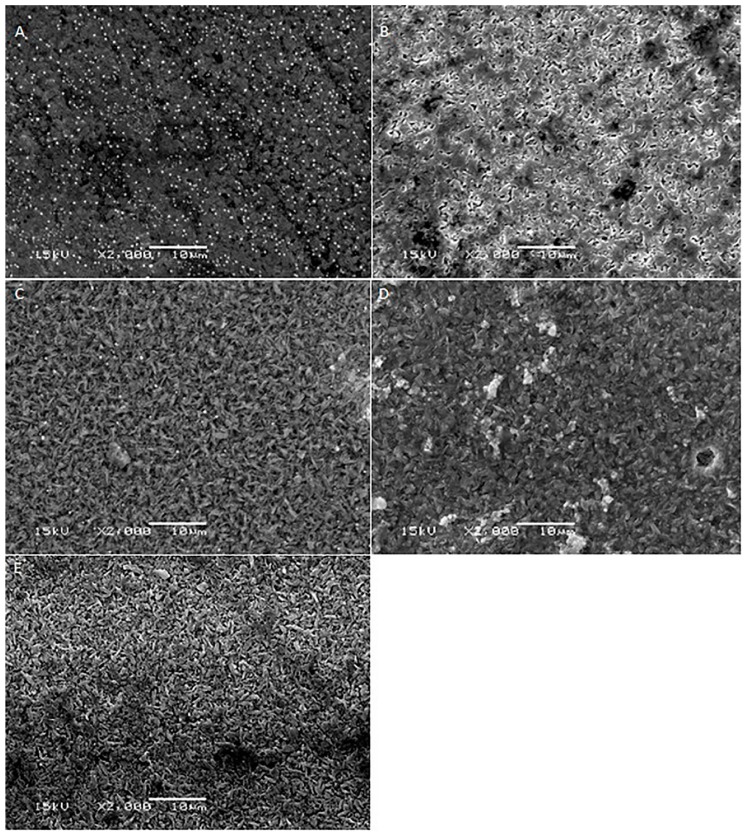
SEM photomicrographs of etched IPS e-max™ ceramic surfaces applied with different etching times. **A**: Control group; **B**: 20 s etching; **C**: 40 s etching; **D**: 80 s etching; **E**: 160 s etching. Original magnification: 2000×; bar = 10 µm.

**Figure 2:**
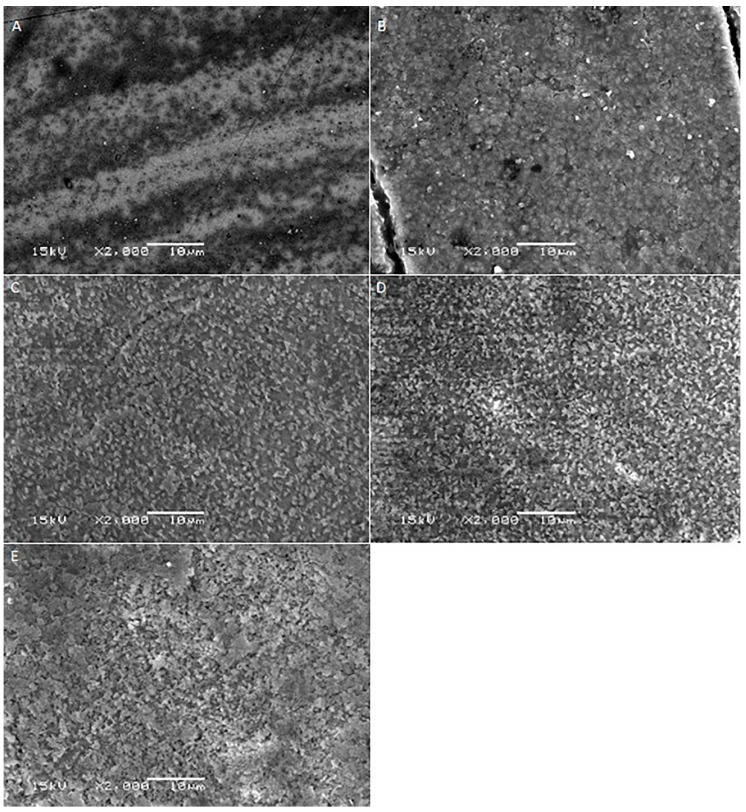
SEM photomicrographs of etched Dentsply Celtra™ ceramic surfaces applied with different etching times. **A**: Control group; **B**: 20 s etching; **C**: 40 s etching; **D**: 80 s etching; **E**: 160 s etching. Original magnification: 2000×; bar = 10 µm.

**Figure 3:**
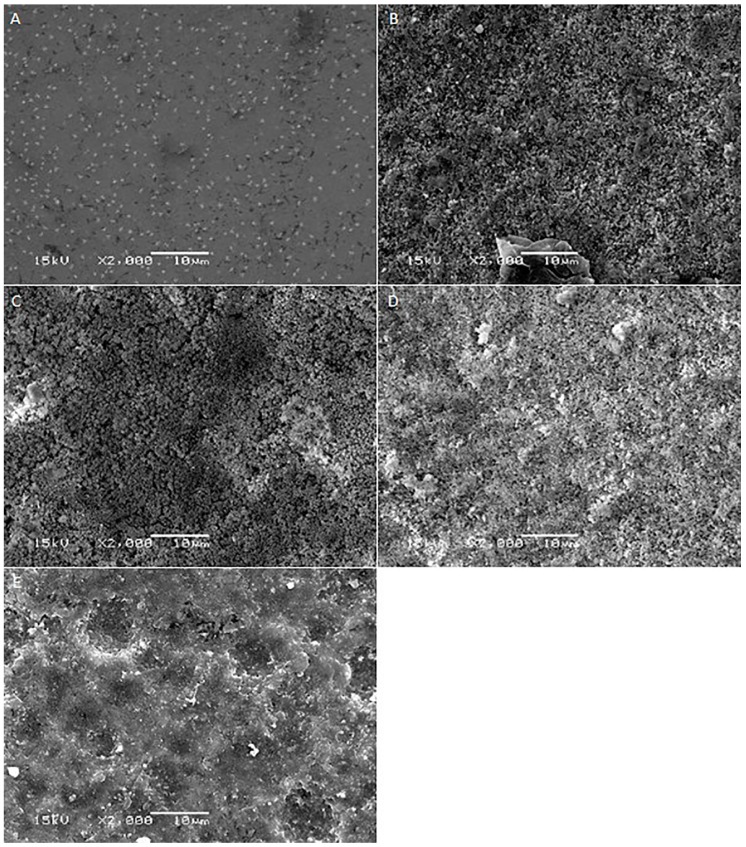
SEM photomicrographs of etched Vita Suprinity™ ceramic surfaces applied with different etching times. **A**: Control group; **B**: 20 s etching; **C**: 40 s etching; **D**: 80 s etching; **E**: 160 s etching. Original magnification: 2000×; bar = 10 µm.

**Figure 4:**
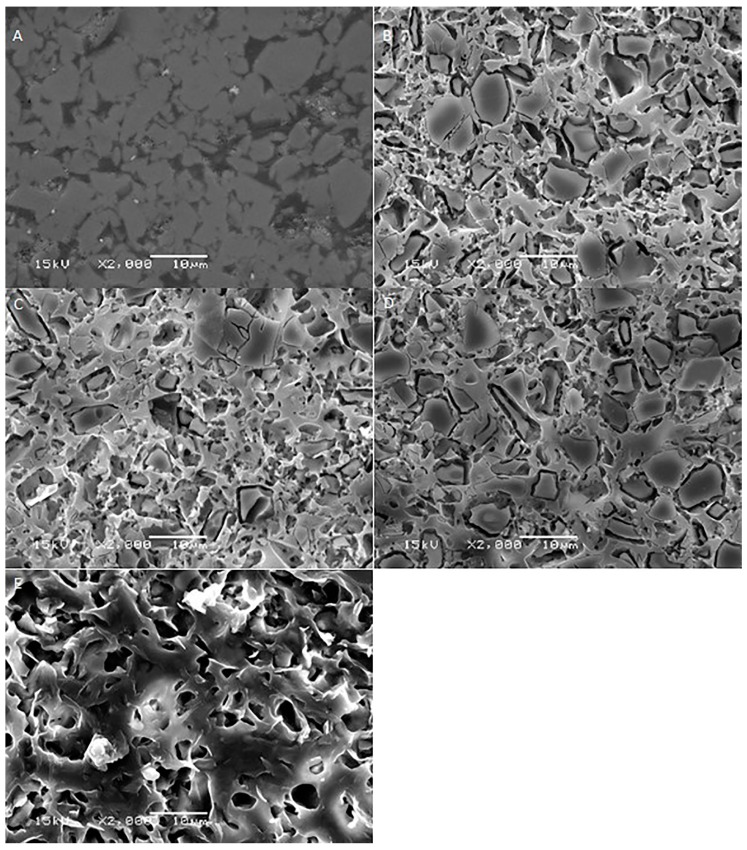
SEM photomicrographs of etched Vita mark II™ ceramic surfaces applied with different etching times. **A**: Control group; **B**: 20 s etching; **C**: 40 s etching; **D**: 80 s etching; **E**: 160 s etching. Original magnification: 2000×; bar = 10 µm.

**Figure 5:**
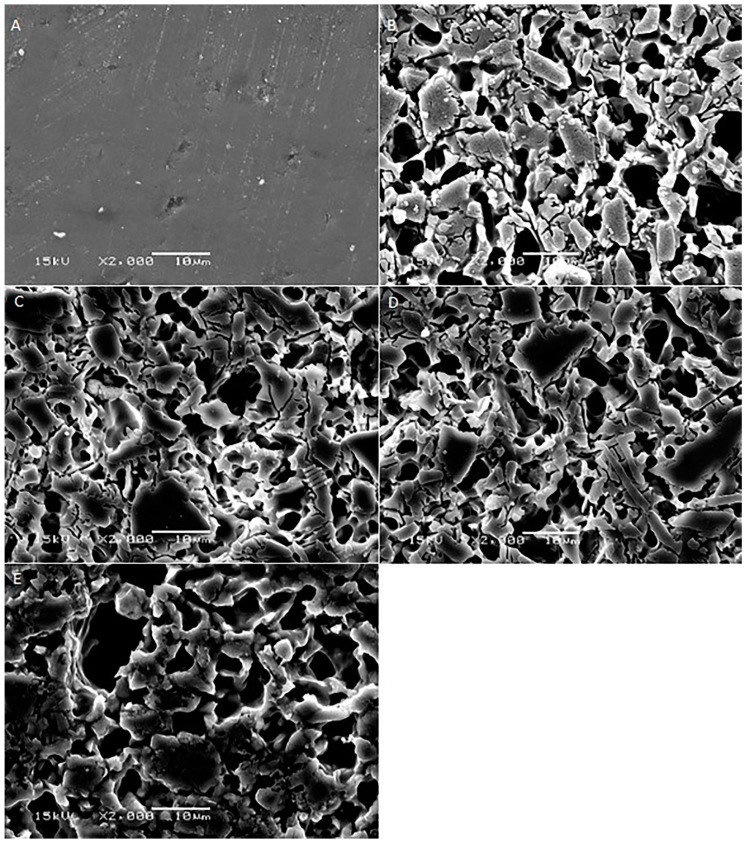
SEM photomicrographs of etched Vita Suprinity FC™ ceramic surfaces applied with different etching times. **A**: Control group; **B**: 20 s etching; **C**: 40 s etching; **D**: 80 s etching; **E**: 160 s etching. Original magnification: 2000×; bar = 10 µm.

**Figure 6:**
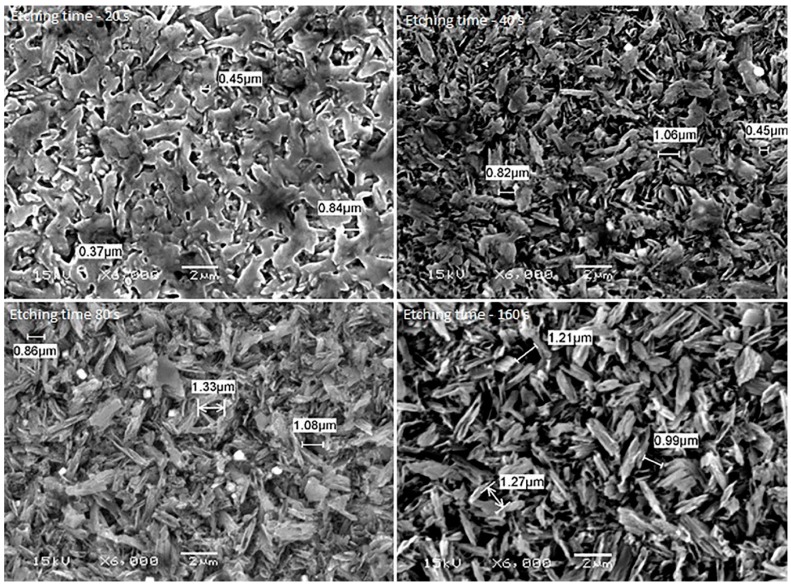
SEM photomicrographs showing microstructure of ceramics etched for different etching times (from the left, upper line: 20, 40, 80 and 160 s). Original magnification 6000×; bar = 2 µm.

**Figure 7:**
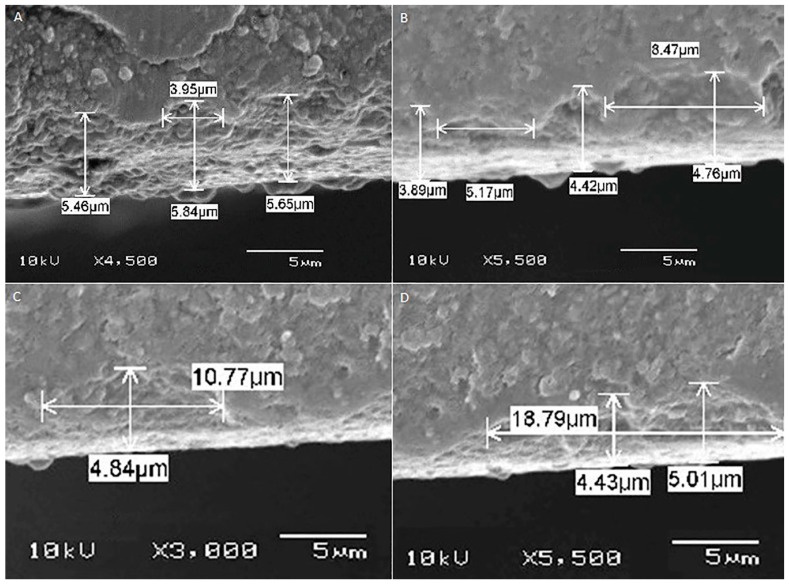
SEM photomicrographs of cross section showing width and pore pattern at different etching times. **A**: =20 s; **B**: =40 s; **C**: =80 s; **D**: =160 s; original magnifications: 4500×; 5500×; 3000×; and 5500×, respectively.

Figure 1, Figure 2, Figure 3, Figure 4 and Figure 5 shows the SEM images of control group and four experimental groups of all the tested materials. The control group specimens showed smooth and homogeneous surfaces without any porosity as a result of polishing. The control group specimen surfaces presented dark and light areas, which represented the glass matrix and crystals (Figure 1A, Figure 2A, Figure 3A and Figure 4A), uniformly distributed fluorapatite and zirconia crystals in lithium disilicate and zirconia-reinforced lithium silicate ceramics (Figure 1A, Figure 2A, Figure 3A and Figure 5A). The etched surfaces showed significant changes in the surface microstructure with an increase in the etching time. The etched surfaces of all specimens were irregular and were characterized by the presence of numerous micro porosities, grooves, and striations as a result of the dissolution of the glassy phase. The specimens etched for 20 s predominantly showed a glassy phase and isolated small pores with closed and irregular borders measuring 0.37 to 0.84 µm (Figure 6). With an increase in the etching duration to 40 s, a weaker glassy phase (SiO_2_, LiO_2_, K_2_O, Al_2_O_3_, P_2_O_5_, ZrO_2_, and HfO_2_) around the crystals that has higher solubility to acid action than lithium disilicate crystal phase dissolved at a faster rate than the crystals (LiS_2_O_5_), resulting in an increase in the size of the pores, which appeared as elongated grooves. A further increase in the etching duration to 80 and 160 s resulted in specimens predominantly showing irregularly oriented crystals measuring 2.56 to 2.97 µm and scratch-like gaps because of an extensive loss of the glassy phase and areas with grain pullout (Figure 6) in particle filled ceramics (IPS e-max™, Dentsply Celtra™, Vita Suprinity™, Vita Suprinity FC™). Specimens of Vita mark II™ (feldspathic porcelain) and Vita Suprinity FC™ (zirconia-reinforced lithium silicate, fully crystallized) showed large cavitations, the dimensions of which increased with an increase in the etching time (Figure 4 and Figure 5). Images of Vita mark II™ typically showed a honeycomb-etched pattern because of the absence of crystal phase, which was different compared to other particle-filled samples (Figure 4). Figure 7 shows the pore width and depth: the pore width and depth increased with an increase in the etching time, the depth ranged from 3.89 µm (Figure 7A) to 5.01 µm (Figure 7D), and the width ranged from 3.95 µm (Figure 7A) to 18.79 µm (Figure 7D).

Table 1 and Table 2 present the mean and standard deviation values of *S*_a_ and contact angle (CA), respectively.

**Figure 8:**
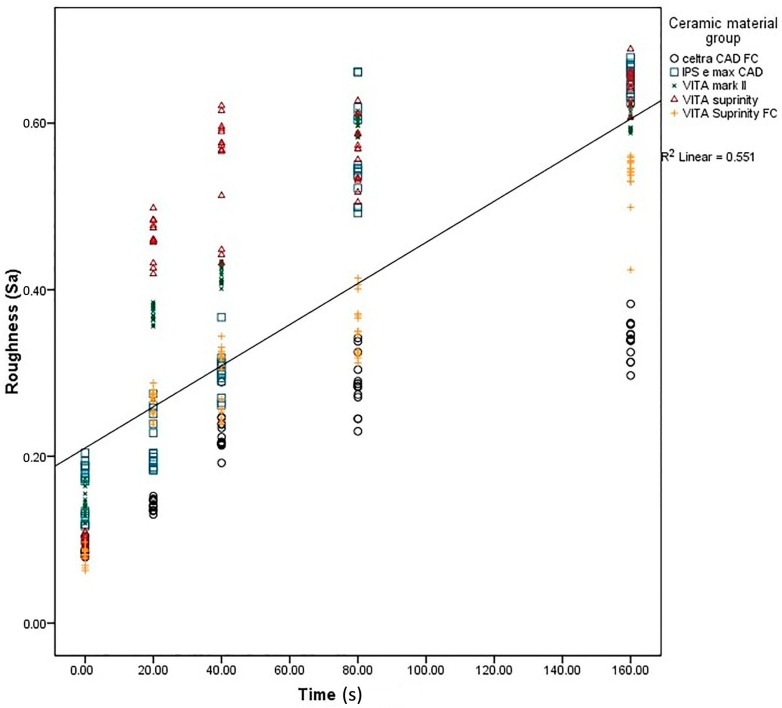
Regression analysis of surface roughness at different acid etching durations.

Results of the one-way ANOVA for *S*_a_ showed a statistically significant difference among the groups (Table 1). The *S*_a_ increased with an increase in etching time in all the materials and experimental groups (Figure 8). Control group, Dentsply Celtra™ and Vita Suprinity FC™ specimens showed the lowest surface roughness (0.08 ± 0.008) and Vita mark II^TM^ showed the highest surface roughness (0.14 ± 0.016). No significant difference was observed between Vita mark II^TM^ and Vita Suprinity™ in 80, 160 s and 40 s, 80 s, respectively (values marked with similar superscripted letters in Table 1).

**Figure 9:**
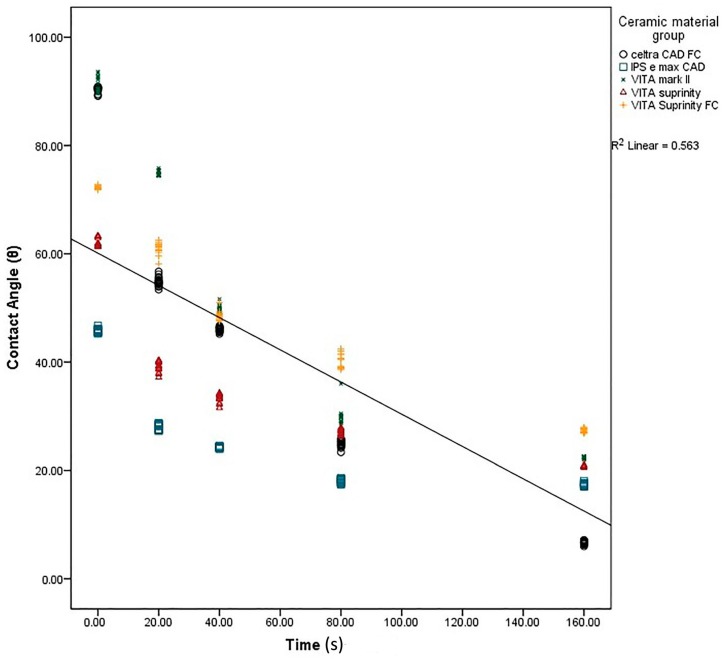
Regression analysis of contact angle at different acid etching durations.

**Figure 10:**
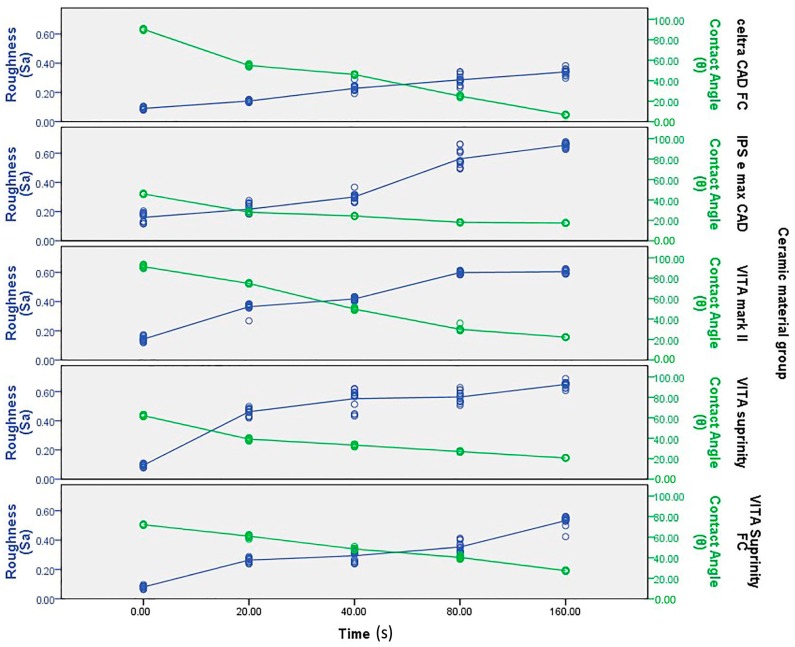
Regression analysis of surface roughness and contact angle at different acid etching durations.

The results of wettability showed a statistically significant difference (a decrease) in CA (an increase in surface wettability) with an increase in the etching time (Figure 9). The samples etched for a longer time (160 s) showed the lowest mean CA, ranging between 6.86 ± 0.35 (Dentsply Celtra™) and 27.4 ± 0.5 (Vita Suprinity FC™) (Table 2). The S_a_ and CA values showed significant dependency on each other in all the materials and at all the intervals (Figure 10).

## 3. Discussion

This laboratory study investigated the effect of the etching duration on the micromorphology, roughness, and wettability of five glass-ceramic based materials. The concentration of the etching acid, HF, has little to do in the clinical scenario because it is pre-determined by the manufacturer, whereas the etching duration is in operator control and can greatly vary, resulting in an increase in surface roughness. Etching of intaglio surface before cementation is an imperative step for better clinical performance of ceramic restorations [25], etching alters surface topography by creating micro- and nano-scale porosities of varying depth and width [26]. The resultant-altered topography increases the surface area for micromechanical bonding (interlocking, retention) with resin composites [27,28,29]. However, creating a desired surface topography for durable bonding depends on the concentration of etchant [4,5,12] and the etching duration [11,30]. Furthermore, the surface roughness also affects wettability of the ceramic surface and thereby the subsequently applied silanes coupling agent and resin composite. The results of this study displayed a significant change in the surface micromorphology, roughness, and wettability with an increase in the etching duration in all the materials tested; therefore, the tested null hypothesis is rejected. In other words, an increase in the etching time resulted in increased wettability or hydrophilicity. The CA values also showed a significant dependence on the values of *S*_a_.

The current study showed a marked change in the surface micromorphology in terms of pores and grooves pattern, width, and depth. This is considered as critical for bonding ceramics to tooth structure with resin composites, and these observations are in agreement with some previous studies [4,5,27,31,32,33,34]. Nagai *et al.* studied the effect of hydrofluoric acid etching on the bond strength of resin composites to lithium disilicate ceramics and reported improved bond strength. The study attributed the improved bond strength to the roughened surface after etching [35]. Zarone *et al.* [36] and Aboushelib *et al.* [37] also showed micro-morphological changes after the etching of ceramic samples, which improved bond strength. Some studies have stated that the correct choice of resin cement and surface treatment procedure suitable for the type of ceramic is a pre-requisite for improving bond strength [36,37]. Etching with hydrofluoric acid selectively dissolves the surface containing a glass matrix and crystals, resulting in the highest bond strength [12]. In the current study, etching for a short duration of 20 s resulted in the dissolution of the glassy phase predominantly around the crystals and creating small isolated pores and fissures. It is noteworthy that there is no real, serious alternative to HF etching [24]. Some other etching agents for porcelain, such as acidulated phosphate fluoride (APF) and ammonium hydrogen difluoride, have been attempted [26]. However, a study conducted by Poorzamani *et al.* showed higher bond strength with hydrofluoric acid-etched ceramic samples than APF gel-etched samples [38]. In the current study, the hydrofluoric acid treatment of ceramic surfaces for 40 and 80 s resulted in the further loss of the glassy phase around the crystals, which exposed the crystal structure. The SEM images presented topographies predominantly consisting of platelet shaped lithium disilicate and lithium orthophosphate crystals (Figure 6). Prolonged etching for 160 s dissolved the matrix around the crystals, which at this stage appeared as protruding out of the glass matrix. Figure 6 (80 and 160 s etching time) shows the typically elongated, randomly oriented pillars of plate-like lithium disilicate crystals measuring 2.56 to 2.97 µm. This suggests that the matrix dissolves at a faster rate than do crystals, and deeper and wider pores are formed when the surface was exposed for longer etching cycles.

Acid etching is the most commonly employed technique compared to grit-blasting to improve the bond strength (adhesion strength). Etching increases the surface area by creating micropores into which uncured flowable resin penetrates to provide durable micromechanical interlocking [39,40]. Etching also cleans the surface by removing debris and impurities. However, prolonged etching does not significantly increase the bond (adhesion) strength. Some studies have reported a reduction in the shear bond strength on prolonged acid etching, which may be due to the smaller pores and fissures that offer better mechanical interlocking sites than do the wider pores [26,41]. Creating an adequately porous surface is a vital step for durable cementation of the indirect restoration, and this can be achieved by etching the surface for shorter etching cycles. However, the etching duration is always governed by the composition of ceramics. Thus, in the present study, a desirable porous surface for lithium disilicate ceramics was achieved by etching for 20 s.

Etching porcelain with hydrofluoric acid is a gold standard because it creates a rough surface required for micromechanical retention with resin composites [11,12,42]. Posritong *et al.* showed an altered microstructure of IPS e-max™ glass ceramic and ZirPress™ after etching the surface for different etching times and reported a significant change in flexural strength [43]. Similarly, Sato *et al.* found a significant increase in flexural strength of 5% hydrofluoric acid-etched lithium disilicate ceramics [44]. The surface roughness is a result of the formation of numerous porosities and grooves due to the acid action on the matrix and the crystal structure. The absolute amount of roughness required for ideal bonding is actually not known, but a positive correlation between roughness and etching duration as observed in the present study has been found [4,11,30,45]. Some studies have also found a positive correlation between roughness and bond strength [11,45]. However, over etching can lead to the formation of large and deep porosities (Figure 7) and may affect the bond strength [20,30,42]. Hooshmand *et al.* reported a significant reduction in biaxial flexural strength after etching the IPS empress and IPS empress 2 ceramics for 2 min [46]. Furthermore, the present study found that the width of the porosities increased at a faster rate than did the depth after having been exposed to longer etching cycles, which resulted in relatively large, wide, and shallow pores. The increase in the pore width is because of the dissolution and disintegration of the weaker glassy phase at a faster rate than the crystal phase. This significant loss of glassy phase weakens the ceramic and affects the bond strength [20,30]. Etching for as low as 20 s resulted in higher mean *S*_a_ values than those for the control group specimens. SEM images showed small pores with a large amount of glassy phase (Figure 6).

Wettability is another important factor crucial for the ultimate bond strength of ceramics to resin composites. In a clinical scenario the fabricated ceramic restoration is bonded to natural dentin (or enamel) using resin composite cement, which is hydrophobic in nature. However, in the present study, the ceramic surface was tested for wettability using deionized water that is obviously hydrophilic. The rationale behind using hydrophilic water was that the etched ceramic surface is treated first with a hydrophilic activated silane coupling agent before it is bonded with hydrophobic resin composite cement. The present study suggests a positive correlation between the etching duration and wettability: wettability increased significantly with an increase in the etching time because of an increase in the surface free energy [6]. This is usually attributed to the fact that etching removes surface impurities such as oxides and other inorganic and organic debris which makes the surface readily wettable for the subsequently applied silane coupling agent and resin composite cement [26]. This laboratory study also showed a significant and positive correlation between *S*_a_ and wettability. A porous surface with improved wettability allows easy spreading and a reaction of silane and resin composites and thereby results in superior adhesion strength.

## 4. Materials and Methods

### 4.1. Materials and Study Design

The lot numbers and manufacturers’ details of five silica-based glass ceramics used in this study are listed in Table 3. Ceramic surfaces were exposed to acid etching for four different time intervals with 5% hydrofluoric acid gel for dental use (IPS Ceramic etching gel™, Ivoclar Vivadent, Schaan, Liechtenstein). The etched samples were examined under a scanning electron microscope (SEM) (JSM-6360LV, JEOL, Tokyo, Japan) to study the pore pattern, width, and depth. Furthermore, the effect of acid etching duration on S_a_ and wettability was evaluated.

### 4.2. Specimen Preparation

Seventy-five rectangular slices measuring 15 ± 0.25 mm × 11 ± 0.25 mm × 2 ± 0.25 mm were cut from each material using a low-speed diamond wheel saw (Model 650, Ladd research industries, Williston, VT, USA). The sectioned specimens were polished with various, up to 1000 grit silicon carbide papers in a sequential manner and polished with a polishing liquid in order to remove gross surface scratches and irregularities using Labpol 8-12 (Extec, Enfiled, CT, USA). Finally, the specimens were ultrasonically cleaned in distilled water for ten minutes using Qantex 140 (L and R, Kearny, NJ, USA) and then stored in sealed plastic containers.

### 4.3. Specimen Organization and Etching Procedure

Specimens requiring crystallization (IPS e-max™, and Vita Suprinity™) were crystallized in a ceramic furnace before etching. Fifteen samples from each material were randomly assigned to one control group and four experimental groups (*n* = 75). Control group received no etching, the remaining samples of experimental groups were etched for 20, 40, 80 and 160 s with 5% hydrofluoric acid. All the specimens were etched in well in a fume hood in a ventilated laboratory environment with all safety measures to avoid any acid hazard. The acid was rinsed with copious amount of water for 30 s and neutralized using a neutralizing agent for five min (IPS Ceramic Neutralizing Powder™, Ivoclar vivadent, Schaan, Liechtenstein). The specimens were washed in running water for 30 s and ultrasonically cleaned for 5 min using distilled water. All 15 specimens from each group were tested for roughness and wettability. SEM analysis was done for one sample from each group of respective material.

### 4.4. Analysis

To study the surface micro-structure of the pores, the samples were examined under a scanning electron microscope (SEM; JSM-6360LV, JEOL, Tokyo, Japan) at ×2000 and ×6000 magnification, respectively. To measure the pore depth and width, etched samples were fractured vertically from the edge of an un-etched surface using a sharp knife with a single hammer impact. The junction of etched surface and the fractured edge was examined, and the depth and width of the pores were measured directly in SEM using image measurement software after tilting the specimen by 45 degrees.

The *S*_a_ was measured using a non-contact optical profilometer [47] (Bruker Contour GT-K, Bruker, Berlin, Germany). This system utilizes a nano lens AFM module with a fully automated turret and platform which is programmable in the *X*, *Y* and *Z* directions. The profilometer delivers a laser light in order to guide the operator to select the test area on the specimen. Measurements on four randomly selected areas on each sample were taken, and their mean was calculated and considered as the final *S*_a_ value. To perform the measurement, the sample was placed on the platform in such a manner that the measuring surface was perpendicular to the optical beam. The measurement area of 1.261 mm × 0.946 mm × 500 µm in the *X*-, *Y*-, and *Z*-axes were scanned with a resolution of 100 points/mm after the application of a Gaussian regression filter with a short wavelength pass. The long cutoff wavelength was 0.08 mm(*X* and *Y*), the short cutoff wavelength was 0.25 mm (*X* and *Y*), and the images were captured in 3× optical zoom using integrated “Vision64” software (Bruker Corporation: Billerica, MA, USA). Several areal surface texture parameter values were measured to characterize the surface topography, which includes *S*_a_ (surface roughness), *S*_p_ (the maximum surface peak height), *S*_v_ (maximum valley or pit depth), *S*_z_ (the sum of largest peak height value and largest pit or valley depth), *S*_q_ (root mean square value of the sampling area), S_sk_ (the measure of skewness or symmetry in roughness), and *S*_ku_ (kurtosis of topography height distribution)/(the spread of height distribution). Mean *S*_a_ was considered in this study because this parameter gives the arithmetic mean of the heights and depths of the sampling surface.

The surface wettability was evaluated by CA measurement using sessile drop technique [47] (Oneattension, Biolin Scientific, Espoo, Finland). One reading was taken per sample from each group. In the analysis, a 2-µL drop of deionized water was dispensed on to the test surface, and the CA measurements were taken. The dynamic CA was measured with built-in software from the time of initial contact of the water droplet, and the images were captured using a built-in digital camera.

### 4.5. Statistical Analysis

The mean *S*_a_ and CA were analyzed using the one-way ANOVA and the *post hoc* Tukey’s test, and the level of significance was 0.05. The linear regression analysis was used to determine the correlation between the etching duration and *S*_a_, the etching duration and CA, and the *S*_a_ and CA.

## 5. Conclusions

The following conclusions were drawn:

(a) An increase in the hydrofluoric acid etching duration significantly alters the surface microstructure and increases the surface roughness and wettability of silica based ceramics;

(b) etching for longer times results in an increased number and width of the pores (Figure 6), and the width of the pores increases at a faster rate than does the depth (Figure 7);

(c) there is a positive correlation between the surface roughness and wettability: an increase in the surface roughness improves wettability.

Further research to perform and analyze the adhesion of ceramics that are etched for different time durations to enamel is recommended.

## Figures and Tables

**Table 1:** Comparison of mean surface roughness (*S*_a_) among different experimental groups.

Materials	Mean Surface Roughness (Mean ± SD)					*p*-Value
	Control Group	20 s	40 s	80 s	160 s	
Celtra	0.08 ± 0.008 ^a^	0.14 ± 0.006 ^b^	0.22 ± 0.02 ^c^	0.28 ± 0.03 ^d^	0.33 ± 0.020 ^e^	0.001 *
e-max	0.16 ± 0.030 ^a^	0.21 ± 0.030 ^b^	0.30 ± 0.02 ^c^	0.56 ± 0.05 ^d^	0.65 ± 0.016 ^e^	0.001 *
Mark II	0.14 ± 0.016 ^a^	0.36 ± 0.020 ^b^	0.41 ± 0.01 ^c^	0.59 ± 0.01 ^d^	0.60 ± 0.010 ^d^	0.001 *
Suprinity	0.09 ± 0.010 ^a^	0.46 ± 0.020 ^b^	0.55 ± 0.06 ^c^	0.56 ± 0.03 ^c^	0.64 ± 0.010 ^d^	0.001 *
Supri FC	0.08 ± 0.009 ^a^	0.26 ± 0.010 ^b^	0.29 ± 0.03 ^c^	0.35 ± 0.03 ^d^	0.53 ± 0.030 ^e^	0.001 *

Key: SD = standard deviation; test applied: one-way ANOVA with the *post hoc* Tukey test. The one-way ANOVA: * indicates statistically significant at *p* < 0.05. The *post hoc* Tukey test: values with different letters superscripted vary significantly.

**Table 2:** Comparison of mean contact angle among different experimental groups.

Material	Mean Contact Angle (Mean ± SD)					*p*-Value
	Control Group	20 s	40 s	80 s	160 s	
**Celtra**	90.33 ± 0.81 ^a^	54.94 ± 1.03 ^b^	46.06 ± 0.45 ^c^	24.86 ± 0.83 ^d^	06.86 ± 0.35 ^e^	0.001 *
**e-max**	45.80 ± 0.56 ^a^	27.95 ± 0.52 ^b^	24.06 ± 0.25 ^c^	18.00 ± 0.37 ^d^	17.53 ± 0.51 ^d^	0.001 *
**Mark II**	91.27 ± 1.43 ^a^	74.80 ± 0.56 ^b^	49.87 ± 0.83 ^c^	30.00 ± 1.73 ^d^	22.20 ± 0.41 ^e^	0.001 *
**Suprinity**	62.13 ± 0.74 ^a^	39.13 ± 0.91 ^b^	33.27 ± 0.79 ^c^	27.07 ± 0.59 ^d^	20.93 ± 0.25 ^e^	0.001 *
**Supri FC**	72.20 ± 0.41 ^a^	61.13 ± 0.18 ^b^	48.67 ± 0.81 ^c^	40.27 ± 1.33 ^d^	27.40 ± 0.50 ^e^	0.001 *

Key: SD = standard deviation; Test applied: one-way ANOVA with the *post hoc* Tukey test. The one-way ANOVA: * indicates statistically significant at *p* < 0.05. The *post hoc* Tukey test: values with different letters superscripted vary significantly.

**Table 3:** Ceramic materials used in the study.

Product	Lot No.	Description	Manufacturer
Celtra	18017131	Zirconia-reinforced (10% by weight) lithium silicate ceramic	DeguDent, Hanau, Germany
e-max	T10332	Lithium disilicate glass ceramic	Ivoclar Vivadent, Schaan, Liechtenstein
Mark II	43660	Feldspathic ceramic	VITA Zahnfabrik, Bad Sackingen, Germany
Suprinity	45960	Zirconia-reinforced (10% by weight) lithium silicate ceramic	VITA Zahnfabrik, Bad Sackingen, Germany
Suprinity FC	47860	Zirconia-reinforced (10% by weight) lithium silicate ceramic	VITA Zahnfabrik, Bad Sackingen, Germany

## Abbreviations

The following abbreviations are used in this manuscript:

CAD/CAM: computer-aided design/computer-aided machining
SEM: scanning electron microscope
S_a_: surface roughness
CA: contact angle
ANOVA: analysis of variance
